# Stressful life experience of the first married women in polygamous families in Gedeo zone, South Ethiopia: a qualitative study, 2021

**DOI:** 10.1186/s40359-022-00753-4

**Published:** 2022-02-22

**Authors:** Nebiyu Mengistu, Seid Shumye, Tinsae Shemelise Tesfaye, Sleshi Haile, Yesuneh Bayisa, Solomon Yimer, Moges Tadesse, Tesfalidet Markos, Derebe Madoro, Dawit Getachew Assefa, Wondwosen Molla, Lulu Abebe, Alemayehu Molla, Aregahegn Wudneh, Bereket Duko

**Affiliations:** 1 Department of Psychiatry, Dilla University, P.O. Box (DU): 419, Dilla, Ethiopia; 2 Department of Anesthesia, Dilla University, P.O. Box (DU): 419, Dilla, Ethiopia; 3 School Medicine, Dilla University, P.O. Box (DU): 419, Dilla, Ethiopia; 4 Department of Nursing, Dilla University, P.O. Box (DU): 419, Dilla, Ethiopia; 5 Department of Midwifery, Dilla University, P.O.Box (DU): 419, Dilla, Ethiopia; 6 Curtin School of Population Health, Curtin University, Perth, WA Australia; 7 School of public health, Dilla University, P.O.Box (DU): 419, Dilla, Ethiopia

**Keywords:** Women, Polygamy, Stressful life experience, Qualitative study and Gedeo zone

## Abstract

**Introduction:**

Polygamy is commonly referred to as the union of a man with multiple women or the practice of having more than one wife at a time. In Ethiopia, polygamy has practiced in all regions. In particular, the stress of polygamous family life predisposes mothers to psychological problems. Being a serious public health issue, the stressful experience among polygamous women was not known in Ethiopia.

**Aim:**

To explore a stressful life experience among first married polygamous women in Gedeo Zone, South Ethiopia, 2021.

**Methods:**

This study was conducted using a phenomenological study approach from February 20–30, 2021. A purposive sampling method was used and an in-depth interview was conducted. Data were collected from 13 first married women from polygamous.

**Findings:**

Three themes emerged from the study including reaction to polygamy, socio-economic challenges in polygamy, and bonds of families in polygamy families. The finding indicated that the status of life experience among first married women in a polygamous family was stressful. They experienced various degrees of psychological difficulties including anger, mistrustfulness, emotional distress, loneliness, emptiness, unhappiness, and lack of intimacy with their husbands.

**Conclusion and recommendations:**

This study highlighted how polygamy is a complex issue and common practice in the Gedeo zone. There has to be a mechanism for serious follow-up to educate women properly. A long-lasting measure to empower women in the economy, social, political, and creating a level of consciousness to resist polygamy is important.

## Introduction

Polygamy is commonly referred to as the union of a man with multiple women or the practice of having more than one wife at a time [1]. It is legally and widely practiced in 850 societies across the globe [2] and it is accepted by a wide range of non-western ethnic and religious groups [3]. Many studies suggest clear advantages where child mortality rates are high and where there are more women than men of marriageable age [4–6]. In many regions, polygamy considered a way to ensure a family’s socioeconomic security and stability [6, 7].

African polygamy is contested in defense of the rights of women increasingly [8, 9]. Nowadays, polygamous marriage in Africa is declining due to the increased cost of living, an increase in women's education, and a gradual change in the status of women to resist polygamy. While some intellectuals appreciate the decline in the practice of polygamy, others are much dissatisfied with the trend [4].

Regarding Ethiopia, polygamy is practiced in all regions while the prevalence varies. Ethiopian Demographic and Health Survey (EDHS) report of 2011, 11% of married women in Ethiopia are in bigamous marriages, with 9% having one co-wife and 2% having two or more co-wives [10]. Similarly, 5% of married men in Ethiopia live in a bigamous marriage having two or more wives [11].

The 2016 EDHS report also shows a similar prevalence of polygamy compared to that of the 2011 report. However, the percentage of women in a polygamous union has declined slightly over time; from 14% in 2000 and 12% in 2005 to 11% in both 2011 and 2016 [10].

Women in polygamous families are commonly unhappy, and the addition of a second or third wife is typically very distressing to the “senior wife.” [11–13] that is, polygamous marriages are more likely than monogamous marriages to be torn by spousal conflict, tension, and jealousy [14–16]. In particular, the stress of polygamous family life predisposes mothers and children to psychological problems [2, 17].

In addition, most women in polygamous societies are unemployed and thus are economically dependent on their husbands or families. Because they cannot support themselves through work, they feel the pressure to marry into a polygamous family as a solution to their economic needs [2, 18, 19].

With few alternative sources of income, they are unlikely to seek another form of marriage, and so many of them remain with their children in a polygamous marriage. However, the mother’s distress has implications for her children. Because it can diminish the level of caring, supervision, and involvement. Some distressed mothers can become withdrawn, depressed, and even hostile towards their children [17, 20, 21]. Some researchers have investigated the psychological impact of polygamy in many countries and reported a significantly greater percentage of co-wives in the inpatient psychiatric population compared with the general population [22]. The findings revealed a significant and increasing prevalence of psychological problems in such women. The most prevalent mental problems in these women were somatization, interpersonal sensitivity, depression, anxiety, hostility, paranoid ideation, and psychoticism [23–26].

Moreover, most researches indicate significant prevalence of mental health issues and its implications in polygamous women [22–26]. However, the relationship between polygamy and stressful life experiences has not been adequately investigated in Ethiopia. A few studies conducted in Ethiopia were mainly cross-sectional surveys focusing on the magnitude of the problems; they do not provide enough details to understand the stressful experience that occurs in polygamous women. However, to the best of our knowledge, there are no any qualitative studies had focused on stressful life experiences among first married women in polygamous marriages., Thus, qualitative research that explore on stressful life experiences in polygamous marriages is key to tackling this issue comprehensively. Therefore, this study has employed qualitative methods to obtain a detailed and full-fledged understanding of polygamous marriage. This qualitative research could contribute relevant viewpoints to generating a more or less comprehensive framework and it will help in filling research gaps in the prevailing discourse on stressful life experience for women in polygamous marriage. The study may also provide useful information for improving the management on mental health implications for women in polygamous.

## Methods and materials

### Study setting

Gedeo Zone, located in Southern Ethiopia, was the study area. The study period was from February 20–30, 2021. The administrative center of the Gedeo Zone is located on the main road from Addis Ababa to Nairobi. According to the 2007 census conducted by the Central Statistical Agency of Ethiopia, this zone has a total population of 847,434, of whom 424,742 are men and 422,692 women. Gedeo zone has an area of 1,210.89 square kilometers and a population density of 699.84. While 107,781 or 12.72% are urban inhabitants, a further 39 individuals are pastoralists. A majority of the inhabitants said they were Protestants, with 73.21% of the population reporting answers in that category, while 10.67% practiced Ethiopian Orthodox Christianity, 7.96% observed traditional religions, 2.44% were Muslim, and 2.11% embraced Catholicism. The zone has one referral hospital which has psychiatric outpatient and inpatient services.

### Study approach

A qualitative descriptive phenomenological study approach was employed, using in-depth interviews because little is known about stressful life experience among senior wife in polygamous family. The purpose of a phenomenological approach in this research is to clarify and enlighten how people understand and comprehend the stressful life experience of first married women in a polygamous family. The in-depth interviews were aimed at getting a greater depth of response as the stressful experience of polygamy is a complex and sensitive subject.

### Participant’s recruitment

Purposive sampling was used to sample study participants, whereas the snowball sampling technique was used to find the subjects. For two reasons, we relied on the help of two recruiters. Firstly, it was impossible for an individual to locate and invite the desired number and type of study participants. We also felt one individual was likely to call a similar but biased group of individuals who probably would be easier for her to convince to come. We were also advised on the best way to locate key people and, through them, approached resource people who helped in the recruitment and/or participated themselves. The following broad data-generating question was used to begin the interview: "How do you feel about your marriage?" Probe Did he ask for permission? Tell me what it has been like to be a wife in a marriage with other wives. The participants responded with a full description, and a few subsequent probes or clarifiers were required.

### Inclusion and exclusion criteria

Subjects included in this study were first married women in polygamous family those who were lived for at least six months in Gedeo zone, whereas those who were critically ill during the data collection period were excluded from the study.

### Data collection and procedures

Six researchers conducted all interviews in Amharic and each interview averaged 40–50 min. An open-ended topic guide was prepared first in English and then translated into Amharic. With some modifications, a topic guide was produced in Amharic to facilitate in-depth interviews. So, we conducted in-depth semi-structured interviews. After the first interview, we identified the next participant(s) by means of the snowball sampling method. The number of participants enrolled was based on data saturation, that is, data collection was terminated once no new themes were identified and a theoretical end point of saturation was reached. Ultimately, a total of 13 participants were recruited. Detailed demographic information of participants is displayed in (Table 1). All participants were subsequently numbered during the process of data analysis to aid in organization and analysis. In addition, all interviews were audio-recorded and were conducted with a list of pre-determined open-ended questions as shown in (Table 2). The audio records of all interviews were transcribed verbatim in Amharic by other researchers unfamiliar with this study. It was conducted in the homes of the participants’ preference. All of the participants signed informed consent before the interview.

**Table 1:** Respondent information

Demographic characteristics	Category	Number of participants (13)	Percentage (%)
Age	20–24	2	15.4
	25–29	3	23
	30–34	2	15.4
	35–39	2	15.4
	40–44	1	7.6
	45–49	2	15.4
	≥ 50	1	7.6
Religion	Orthodox	4	30.7
	Protestant	9	69.3
Ethnicity	Gedeo	8	61.5
	Oromo	3	23
	Amhara	2	15.4
Occupational status	House wife	9	69.2
	Governmental	1	7.7
	Non-governmental	3	23
Educational status	Unable to write and read	8	61.5
	Primary and secondary	4	30.7
	College and above	1	7.7
Family size	< 3	3	23
	3–6	4	30.7
	> 6	6	46.1
Number of co-wives	One co-wife	6	46.1
	Three co-wives	3	23
	Four co-wives	4	30.7
Income	< 2500 ETB	8	61.5
	> 2500 ETB	5	38.5
Residence	Rural	9	69.3
	Urban	4	30.7

**Table 2:** Interview guide

Open-ended questions about Stressful life Experiences among first married polygamous women
1. How do you feel about your marriage? Probe: Did he ask permission? /inform you when he took another wife?
2. How would you describe your relationship with your husband?
3. What does your relationship with other cowives look like?
4. Do you live in the same compound or household? If so, tell me about your daily interactions
5. Are you happy with the work load allocation between your co-wives?
6. How would you describe your relationship with your stepchildren?
7. How would you describe your interaction with society?
8. How do you see the treatment your husband gave you in terms of time allocation? Probe: What about attention and care?
9. Do you think you would be better or worse off if you were not in a polygamy marriage? Probe Why?
10. What issue normally leads you to be stressed?
11. What is the most stressful situation you have faced so far at home? Probe: How did you handle it?
12. Can you describe a time when your stress resulted in conflict at home?
13. Is there anything you would like to add?

### Strategies for improving quality

Lincoln and Guba posit that the trustworthiness of a research study is important to evaluating its worth. Trustworthiness refers to the assessment of the quality and worth of the complete study, while helping to determine how closely study findings reflect the aims of the study, according to the data provided by participants [27].So, Lincoln and Guba’s criteria were used to improve the quality of the. To ensure the acceptability of data accuracy and authenticity, ongoing verifying and coding of data was carried out by help of participants. To determine the dependability, two members of the research team encoded the data separately and reached a high level of agreement. Also the data collection, implementation, and encoding of data were done very carefully and enough time was allocated. In the field of transferability, the gathered information by five experts outside the research team, who are expert on qualitative research, was reviewed and approved. A comprehensive and complete description of the studied subject was also provided.

### Data analysis

After writing the data of interviews, the texts was repeatedly read by the researchers to create more compact semantic units. Then, the data were classified using induction. Coding was performed using NVivo software (version 11). To this end, the extracted codes were classified according to similarities and differences using the continuous comparison method. The classes were then organized in such a way that there was the most internal consistency and the least external incompatibility. Besides, the accuracy and precision of the initial coding was also carried out in a standard process under the supervision of qualified experts in the field. To minimize bias, each of the coders coded the interviews independently and differences were discussed to determine the initial coding framework. To increase the validity of the study, themes and subthemes were reviewed and verified by other researchers. Finally, researchers translated themes, subthemes and representative quotations into English. In addition, quotations were edited to correct grammar or remove content of repeated words and stutters.

## Findings

### Socio-demographics characteristics

The participants involved in this study were thirteen [13] first-married women in polygamous families. The mean age of the participants was 33.4 years (ranging from 20 to 54 years); the average family size was 6.8. Nine of the participants were Protestants, and four were religious adherents of the Orthodox faith. Concerning the number of co-wives, six of the women have one co-wife, another three have four co-wives, and the remaining three have three co-wives in their marriages. Of the thirteen participants, twelve were small-scale market merchants, and one was an employee. Eight of the participants had no formal education, four had primary education and secondary education, and one was a diploma holder as shown in (Table 1).

### Identified themes of first married women in polygamy

The researchers read through all the transcribed data to identify the common themes. Three major themes were identified. This includes reaction to polygamy, socioeconomic challenges in polygamy, and family bonds in polygamy as shown in (Table 3).

**Table 3:** Themes and subthemes about stressful life experiences on first married women in polygamous family

Themes	Subthemes	Codes
socioeconomic challenges in polygamy	Economic barriers	It referring to a lack of resources to live a normal life. It includes food, clothing, shelter, educational materials, and the preservation of land ownership
	Responsibility	Which is any activity that seeks attention or concern regarding the family that demands time, money, and labor work effort, e.g., taking care of the whole family, childbearing, and being exhaustively involved in a work burden
Family bonds in polygamy	Relationship	Any kind of relationship, either positively or negatively, with a husband, co-wives, stepchildren, and society, as well as being trustworthy within the family
	Reproductive issues	Any reference related to reproductive health issues and experiences such as infertility and sexually transmitted diseases. E.g. "My husband had a desire to have another child but I couldn’t give him one, so he married another woman to fulfill his needs"
	Support	**T**he act or process of supporting or helping people to cope with their problems. It could be spiritual, social, and financial support
Reaction to polygamy	Abuse	This can be described as a maladaptive pattern of verbal, physical, and sexual harassment. It also includes mistreatment and discrimination by her and her co-wives
	Alcohol use	It refers to excessive use of alcohol which is up to the level of intoxication
	Legal issues	Any legal action relating to polygamy is referred to as a legal issue
	Psychological issues	It is a condition characterized by abnormal thoughts, feelings, and behaviors

#### Socioeconomic challenges in polygamy

In a polygamous marriage, it is hard to provide all necessities to all family members. Food, clothing, and school supplies, among other things, should be provided equally by the husband for all of his family members. Because the husband cannot provide all these essential resources for all the wives, the wives are obliged to work very hard and do different jobs to cover their daily expenses. Many interviewed women disapproved of polygamous marriages because of financial problems.

One of the participants, a 54-year-old merchant woman with three co-wives, claimed: *"After he married another woman, we struggled to cover our expenses, such as food, shelter, and school fees, so I tried to sell beverages to supplement my income, which wasn't enough. The other wife also complained about the money her husband gave her from the farm profits.”*

In most parts of Ethiopia, the husband is responsible for the provision of essential needs to his wife and children, so the husband needs to work hard to cover the daily expenses but in contrary to this most of our respondents claimed that their husbands spend their time drinking and talking. They anticipate that their wives will cover the expenses for all household matters, but in contrast, strikingly, husbands even take money from their wives.

A 31-year-old merchant mother with three children said: "The burden is difficult. My husband does not work. He also does not go to work most of the time, so he would rather sleep or drink alcohol. As you can see, I sell bread and, on occasion, alcohol. I used to sell firewood too. Currently, I am in an economic crisis. My children and I survive on the money I make from selling bread. Worse, he robs me and gives it to his second wife. Because of this, now I am selling this bread by taking it on credit". *She also complained*: *"I used to lend money to people, but now my daily life is going backward." "Kulkul new yenenuro" in Amharic, which means my life is going downhill.*

Sometimes, the husband may have a favorite wife or an incline towards the new wife. This worsens the economic challenges since he may give the money to his favorite charity. Not only money, but they may also give all their properties, like land, to their favorite one. The condition leads to emotional stress and makes the life of the first woman difficult. On this fact, another 20-year-old housewife with one co-wife claimed that: *"He took and sold every property that we had already. He even demolished our compound's wooden fence, sold it, and gave the proceeds to his co-wife. I was very hurt when he did this."*

In polygamy, there is an unequal distribution of household activities and child-rearing responsibilities between the first married woman, the husband, and the co-wives. The first married lady is expected to serve her husband by cooking, dressing, and taking care of her own needs. She also has to look for the children and meet their requirements, which include education.

A 40-year-old housewife woman with two co-wives explained this situation by saying, *"My husband is very hesitant about important daily activities and things that require more of his involvement." He was doing nothing. He wastes his time by walking around. I provide him with food. If not, he will just sit or go away. Instead, I am exhaustively involved in issues that need help, and I work hard to raise my children. As a result, I believe these things stress me out. "*

Women have faced different difficulties within their polygamous marital relationships. Thus, they need help and support from their families, neighbors, and religious leaders to share their pain, to help them through hard times, and sometimes to solve their problems, like their own. They feel safe and comfortable within this support system. For instance, one of the 33-year-old housewives mentioned earlier said that: *"We quarrel many times, then I will go to my parents, stay there for two or three months, then we will make peace because of mediation by the elderly."*

The elderly and religious leaders have a great role in maintaining peace in a polygamous marriage. Both the wife and the husband will listen to them. Thus, the wife will go to them and tell them about her problems. They will listen patiently, and they will advise both parties and solve the problem. Neighbors are also important when there is a fight or physical abuse because they come in between and save the woman from physical trauma. One of the participants, a 25-year-old housewife with a total family size of nine, complained that *"During the fight, I will go to my neighbors. I will spend the night there and will come back the next morning. Sometimes I will stay for up to four days and then the elders resolve the issue and make me return home."*

Women in polygamous marriages are lonely and need someone to share their burdens with and whom they can trust, but if they do not have such support, they will become depressed and stressed. This was substantiated by a 28-year-old housewife who said that *"Before this second marriage, he used to support me; I lean on him, and he assists me, which makes me feel better." However, now I don’t have any people by my side to help me. I just need someone to say, "I am here for you. I am here for your children." However, losing this causes me the most "stress."*

#### Family bonding in polygamy

Polygamy structures social relationships within the household by requiring cooperation among co-wives in productive (domestic, agricultural) and reproductive (conjugal, childrearing) arenas. The effect of polygamy on children has been researched but is still unclear. Many believe that polygamy is beneficial because it allows men to have many children and helps society. On the other hand, many view polygamy as an inappropriate relationship.

One of the participants, a 29-year-old elementary teacher, tried to express this concept as follows: "I am thinking about leaving my marriage and marrying another person. However, I was worried about my children and couldn’t leave them. That is *how* I feel. In addition to getting married to another woman, my husband wouldn’t do anything properly *around* the house. He simply comes and goes. For these reasons, I didn’t have a good relationship with him. Above all, when I see him, I feel something that I can’t describe*. This suggests that women's standing is connected with their husbands'; there is tremendous societal pressure on women not to divulge personal thoughts about their marriages that could jeopardize their allegiance to the established social order. To put it another way, the aforesaid woman also mentioned her husband's timeshare, stating, "Most of the time, he spends his time with his second wife." He didn't even bother to come up to visit his kids. We don't have sex very often because I don't want it."*

Female fertility is affected by the interplay between marital rank, household status, and cultural norms in polygamous marriages. A 22-year-old housewife woman expressed that: "According to our culture that I know, there are only two conditions in which a second marriage is possible*, and so on."* The first is if the husband has a large farm and a lot of money and the first wife is unable to manage it. Therefore, the husband will tell her that he will be *marrying* a second wife so that she can help her. And, the second condition is *that*, after five years of marriage, if the first woman doesn’t give birth, it is assumed that she approves of the second marriage. My husband, on the other hand, does not even have a regular job, but he married a second wife and keeps her in a rental house*.”*

#### Reaction to polygamy

The most frequently mentioned psychological burden of women in this study was the perceived burden of taking care of the whole family, including income generation, and they are prone to experiencing stressful life events. On top of that, husbands threaten them in different ways, like insulting them, telling them to commit suicide, or telling them they are going to kill them one day. One of the participants, a 27-year-old housewife, described it by saying: "I am so angry that my instinct says go away, go away, leave the children behind, and live your own life*. According to her, she was mentally overwhelmed and unable to deal with her husband because she had lost trust in him after everything that he did to her. She stated that "I started hating him and also, once I took him to court, I decided to abort the case."*

The woman shared nothing with her husband, and no family members from that household talked to him. Because the members of that household believed that women were weak by nature and had to struggle to manage their lives, the women tried to alleviate their problems by believing that God was in control of everything in their lives. Another 45-year-old housewife with eight family members stated that “*she got stressed when she lost her daily job. She feels angry and she claims that her husband faked her whole life. She described her life as: "They (community) tell me that I am damaged, like a fallen tree. However, she decided to leave and start a new life, but they were stuck in the same situation because of her children.*

Common experiences shared by women in polygamous marriages in this particular study were that their husbands disturbed their lives due to their excessive use of alcohol almost every day. Apart from the psychological and social effects, this also aggravates and even exposes the family to economic problems. One of the participants, a 47-year-old merchant woman, described this circumstance as: "My husband always drinks alcohol, such as Tej, Araki… When he drinks, he insults me and tells me not to say *"*Egizahbir in *Amharic"* (meaning God in English)".

In general, within polygamous households, there are many tensions and disputes between the different parties involved: the husband, co-wives, and children. Abuse can range from verbal, emotional, sexual, to physical abuse. The majority of our participants stated that their husband could be abusive, especially if he drank alcohol.

A 25 -year-old woman mentioned that: "When he drinks, he insults me*,* and when I pray to God by calling his name, he becomes angrier and *tells* me not to say "Egizabher Egizabher" in Amharic, meaning don’t say God repeatedly. He comes every Sunday and tells me to commit suicide by holding the electricity, and he shouts at me, saying to get out of his house and *says* he will kill me with his bare hands, and because of this, I am always afraid of losing my life. Furthermore, he always says " (MUCHE' in Amharic, which means "you must die") and other words. These words make me so stressed. He only fought with "Timfash" (in his own words) and never touched or hit me with his hands. When he said these words, I did nothing but leave the house to get away from him."

Despite being illegal, polygamous marriage remains a common practice in Ethiopia, and that poses some obvious problems. One of the participants, a 30-year-old housewife, described this situation by saying: "Everything that he did to me, I started to hate him and also took him to court*."* However, I decided to abort the case. Because it was a stressful situation*,* which can be described as a tension between my value of maintaining my family and the pressure from my neighbors and relatives to get divorced or stop seeing him and his children, *I have* unfortunately decided to stay and maintain the family. Perhaps I will give my permission to get him part of the farm revenue, almost half of it*."*

## Discussion

The findings of current study include three main themes: socio-economic challenges, reaction to polygamy, and family bonds in polygamy of respective spouses are key dimensions of polygamous family.

### Socio-economic challenges

The current study revealed that polygamism affects senior wives economically and psychologically to a greater extent. Women in polygamous marriages are more likely to live in financially vulnerable households. This probably reflects the economic context of polygamy, which transfers a heavy economic burden to the families of polygamous couples. This finding is supported by the study done in Afghanistan [28], West African countries and Uganda [29, 30].

Another striking finding of the present study was the husband’s lack of willingness to take responsibility for the provision of essential needs for his wife and children. For the family to develop supportive relationships, healthy communication, mutual respect, and teamwork are crucial. But in this study, inequality between first married women, husbands, and co-wives was commonly measured by sharing household activities and childbearing responsibilities. This might be due to too much drinking and not working properly. Therefore, the first married women were challenged to cover their expenses, such as food, shelter, and school fees. A study conducted in Nigeria, Ghana, and Israel [31–33] reported that lack of taking responsibility by the husbands affect the senior wife to suffer from socioeconomic challenges.

### Family bonds in polygamy

Based on the results of present study, relationship is also one of the concerns the senior wife in polygamous marriage. The finding is supported by research done in Cameroon [34],Palestine [23], Iran [35] and Turkey [36]. The possible justification might be due to the fact that when the husband enters into a second or subsequent marriage, this is likely to affect their access to resources, attention, and time allocation among the wives. Therefore, competition over access to resources, including the husbands’ time and attention, can contribute to anxiety and tensions between co-wives as they seek to negotiate relative positions and consolidate their influence within the node. Competitive relationships between wives in many polygamous families are common [19, 37, 38].

According to the current study, social support during interviews indicated how they looked for social support from friends, family, and neighbors very bad as participants report. which is supported by studies done in middle-east countries [39, 40] it may be due to the complexity of polygamy that is a product of power relations, with deep cultural, social, economic and political roots [41].

Another factor emerging from study was that for most women, infertility, desire for another child, and fear of divorce are the cited reasons for allowing co-wives in their marriage. A study conducted from Ghana and Cameroon [34, 42–44] explained the same issues. That is may be due to failure of a woman to produce offspring after a respectable amount of time has passed another motivation to take more wives and female fertility is affected by the interplay between marital rank, household status, and cultural norms in polygamous marriages [45, 46].

### Reaction to polygamy

Furthermore, the current study findings indicate that the life of the first married woman in a polygamous family is abusive and that they experience various degrees of psychological difficulties, such as anger, mistrustfulness, a struggle for existence, loneliness, emptiness, unhappiness, and a lack of intimacy with their husbands. The findings of this study back up previous research on polygamous women in Ghana, India, and Germany [42–44].

Another study of Palestinian polygamy families shows that senior wives, lower family functioning, and higher marital distress in a polygamous family may, in turn, exacerbate negative role modeling and impede children’s growth and achievements [47]. Perhaps this is consistent with our findings, which show that first-married women experience stressful life events for a variety of reasons, including an insult from their husband, a negative attitude toward society, shouldering their family issues alone, and a lack of self-assurance.

In particular, first married women face stressful life events due to the psychological burden of loneliness and income generation for their family in this study. This is supported by the study done in Jordan and United Arab Emirates [26, 48], This might be due to the fact that if they do not find that they are unable to feed their family, they will become concerned about finding alternative means of obtaining food and that makes them mentally overwhelmed [49].

### Implications

The issue of polygamous marriage has its own unique circumstances in different cultures and countries, and this qualitative study provided evidence based on the Ethiopian context to illuminate the effects of polygamy on senior wife in polygamous family. Moreover, the stakeholders like psychiatrists and nurses should strengthen psychoeducation regarding stressful life experience in first married women and their families to reduce their misconceptions. Considering the importance of psychiatric community rehabilitation in stressful life experience among polygamy, governments should establish local community-based mental health services. Governments should promote the successful and culturally adapted mental health models to other areas in Ethiopia as well as integrate the mental health workforce at all levels to implement the mental health models efficiently. With the aforementioned effective steps, the future mental health system may improve the stressful life experiences in first married women in polygamous family.

## Limitations of the study

Our research has several limitations. It was conducted with a relatively small sample size from a small area, and thus, it is difficult to generalize the results. But given that we have investigated stressful life experiences among first married polygamous families in both urban areas and rural settings, it may still provide useful information to researchers in the field. Furthermore, it may also provide support for this kind of research in other African countries with a cultural context similar to that of Ethiopia. However, as a qualitative study, there may be limited generalizability beyond our setting. Another limitation is that the present study only looked at the senior wife in a polygamous family and the methods of data collections that is all information was collected through in-depth interview**.**

## Conclusions and recommendations

The current study has identified polygamy as a common type of marriage. The first married women in polygamous marriages face socioeconomic challenges, negative reactions to polygamy, re-productive contributions, and family bonds in the polygamy of their respective spouses, which are key dimensions.

The complexity of the challenges that women within polygamous marriages experience, as well as their decision-making abilities, requires more attention in both qualitative and quantitative research. Practitioners and policymakers need to be aware of the psychological, familial, and economic effects of polygamy on women and their children. According to the findings, polygamous families’ experience more marital distress. It should be noted that this research serves as a voice for women in polygamous marriages and raises the question of the mental health of people where polygamy is practiced. And also, it’s better to educate women properly and that should be the long-lasting measure of empowering them economically, socially, and politically and thereby creating the level of consciousness to resist polygamy. More research is needed to compare women in polygamous marriages based on their order (first, second, and third, etc.), and the effect of polygamous marriages on children's well-being should also be investigated.

## Data Availability

All data generated or analyzed during this study is included in this published article. On reasonable request, the data set for the current study is available from [Nebiyu Mengistu, email: nebiyumen@gmail.com; Mobile: + 251,931,333,504, Dilla University, Dilla].

## Abbreviations

E (DHS): Ethiopia (Demographic Health Survey)
ETB: Ethiopian Birr
LAMIC: Low- and middle-income countries
SSA: Sub-Saharan Africa
UNICEF: United Nations Children's Fund
WHO: World Health Organization
