# Correction: Stressful life experience of the first married women in polygamous families in Gedeo zone, South Ethiopia: a qualitative study, 2021

**DOI:** 10.1186/s40359-022-00898-2

**Published:** 2022-08-02

**Authors:** Nebiyu Mengistu, Seid Shumye, Tinsae Shemelise Tesfaye, Sleshi Haile, Yesuneh Bayisa, Solomon Yimer, Moges Tadesse, Tesfalidet Markos, Derebe Madoro, Dawit Getachew Assefa, Wondwosen Molla, Lulu Abebe, Alemayehu Molla, Aregahegn Wudneh, Bereket Duko

**Affiliations:** 1 Department of Psychiatry, Dilla University, P.O. Box (DU): 419, Dilla, Ethiopia; 2 Department of Anesthesia, Dilla University, P.O. Box (DU): 419, Dilla, Ethiopia; 3 School Medicine, Dilla University, P.O. Box (DU): 419, Dilla, Ethiopia; 4 Department of Nursing, Dilla University, P.O. Box (DU): 419, Dilla, Ethiopia; 5 Department of Midwifery, Dilla University, P.O.Box (DU): 419, Dilla, Ethiopia; 6 Curtin School of Population Health, Curtin University, Perth, WA Australia; 7 School of Public Health, Dilla University, P.O.Box (DU): 419, Dilla, Ethiopia

## Correction to: BMC Psychology (2022) 10:40 10.1186/s40359-022-00753-4

Following publication of the original article [1], the authors flagged that their article had published with an incomplete acknowledgements declaration. Namely, the following text had been erroneously omitted from the declaration: "Finally, we would also like to thank the Health Professionals Education Partnership Initiative Ethiopia (HEPI) for initiating the research and providing training support."

The published article has since been corrected with the complete declaration. The authors apologize for any inconvenience caused.
